# Knowledge of physical activity recommendations in adults employed in England: associations with individual and workplace-related predictors

**DOI:** 10.1186/s12966-015-0231-3

**Published:** 2015-05-23

**Authors:** Emily C. L. Knox, Hayley Musson, Emma J. Adams

**Affiliations:** British Heart Foundation National Centre for Physical Activity and Health, School of Sport, Exercise and Health Sciences, Loughborough University, Loughborough, UK

**Keywords:** Moderate-to-vigorous physical activity, Guidelines, Workplace, Employee, Knowledge, Health

## Abstract

**Background:**

Physical activity guidelines state that adults should engage in at least 150 min of moderate to vigorous physical activity (MVPA) per week to benefit health. A high proportion of adults in England fail to reach this target. Accurate knowledge of MVPA guidelines could influence the amount and quality of MVPA engaged in by adults. This study aimed to determine knowledge of the MVPA guideline within a large sample of working adults in England and identify individual and workplace-related predictors of knowledge.

**Methods:**

10,992 adults completed an online survey which included questions on demographics, knowledge of the MVPA guideline and workplace predictors for physical activity. Multinomial logistic regression identified predictors of underestimating, overestimating or not knowing the MVPA guideline relative to accurately reporting the guideline for males and females separately.

**Results:**

Respondents were 37 % male, 95 % White, 63 % with a degree or higher, and had a mean age of 38.9 ± 11 years. The MVPA guideline was accurately reported by 15 % of adults while 13.8 % overestimated, 8.9 % underestimated and 62.3 % failed to provide any estimate of the guideline. Low education predicted underestimation (females: OR = 0.36, 95 % CI 0.17, 0.80) and not knowing (males: OR = 0.37, 95 % CI 0.14, 0.96; females: OR = 0.36, 95 % CI 0.19, 0.69). Ethnicity was a significant predictor for females only (OR 3.55, 95 % CI 1.46, 8.63; OR 4.03, 95 % CI 1.58, 10.27; OR 3.73, 95 % CI 1.67, 8.33). Employer support for physical activity was a significant predictor of accurate knowledge of the MVPA guideline for both males (underestimation: OR = 0.63, 95 % CI 0.40, 1.00; ‘don’t know’: OR = 0.71, 95 % CI 0.51, 1.00) and females (overestimation: OR = 0.72, 95 % CI 0.53, 0.97; underestimation: OR = 0.66, 95 % CI 0.47, 0.92; ‘don’t know’: OR = 0.60, 95 % CI 0.47, 0.76).

**Conclusions:**

Knowledge of the MVPA guideline within working adults in England is low. Employers should play a role in using targeted strategies to increase knowledge as employer support-related factors may influence knowledge of the MVPA guideline. Employers who assert strategies to promote physical activity and encourage employees who have responsibility for promoting health to educate their colleagues may help improve the MVPA knowledge of their employees.

**Electronic supplementary material:**

The online version of this article (doi:10.1186/s12966-015-0231-3) contains supplementary material, which is available to authorized users.

## Background

Physical activity reduces the risk of morbidity and mortality from chronic diseases [1]. Physical activity guidelines state that adults should engage in at least 150 min a week of moderate-to-vigorous physical activity (MVPA) to obtain health benefits [2–5]. In England, only 67 % of males and 55 % of females meet current physical activity guidelines based on self-report measures [6]. However, objective measurements taken in 2008 using accelerometers suggest that the actual proportion of adults meeting physical activity recommendations in England is much lower at only around 6 % [7].

Knowledge of physical activity guidelines has been theoretically supported as a pre-requisite to individual’s positive motivation to engage in more physical activity [8, 9]. Individuals who know how much MVPA they need to achieve to benefit their health may be more capable of judging whether or not they need to engage in more MVPA and subsequently be more likely to make positive behavioural adjustments [10, 11]. Only one previous study has inspected knowledge of the current (i.e. as of 2011) physical activity guideline for adults. Knox et al. [12] found knowledge of the adult guideline to be 18 % within a highly educated sample of 1797 adults. A better understanding of the factors associated with knowledge of guidelines will benefit attempts designed to promote and increase physical activity levels. Hunter et al. [13] identified that the factors associated with overestimating, underestimating and not being aware of guidelines differ in a sample of adults in Northern Ireland. This has important motivational implications which may impact upon decisions around the strategies which should be employed to improve knowledge.

In recent years a number of studies have been published which identify the workplace as a potentially important setting for the promotion of physical activity [14–16]. There are more than 30 million people in employment in England the majority of who spend 31 h or more at work each week [17]. The workplace therefore is an important setting in which adults can be reached and potentially educated with regard to recommendations for participation in physical activity to benefit health. In addition, employer support has the potential to have an impact on workers perceptions and behaviours towards physical activity. Physical aspects of the working environment such as the availability of showers, policy aspects such as the provision of programs and normative influences of co-workers can all influence physical activity [18, 19]. Employers can also support physical activity in a number of ways such as by implementing health promotion practices [20], sponsoring public transport passes [21] and facilitating changes to workstations which encourage physical activity [22]. Knowledge of physical activity guidelines within working adults has not been previously investigated and the effect of employer promotion of physical activity and sport on knowledge of physical activity guidelines has yet to be explored.

The aim of this study was to identify the prevalence and correlates of knowledge of current physical activity recommendations within a large sample of adults working in England. Further, the hypothesis that employer support for physical activity is associated with knowledge of the MVPA guideline was explored.

## Methods

Data were collected through the *County Sports Partnership Network Workplace Challenge* project which was part of the ‘Get Healthy, Get Active’ initiative funded by Sport England. 16,292 adults from workplaces across England signed up to the challenge between 1^st^ December 2013 and 30^th^ September 2014 and were invited to complete the baseline online survey. Ethical approval for the study was obtained from Loughborough University Ethical Advisory Committee (reference R13-P170).

### Measures

#### Dependent variable

Participants were asked; “Do you know what the national recommendations are for taking part in physical activity, in terms of minutes per week of moderate intensity physical activity?” Participants who responded ‘no’ were labelled as ‘don’t know’. Participants who responded ‘yes’ were then asked “what are the national recommendations for taking part in physical activity, in terms of minutes per week of moderate intensity physical activity?” Answers of 150 min a week which is consistent with current physical activity guideline information were considered correct. Individuals giving answers of greater than 150 min a week were labelled as over-estimators and those giving answers of less than 150 min a week were labelled as under-estimators.

#### Predictor variables

Participants were asked to provide a number of demographic variables including: age, gender, highest educational qualification, job type and employment status. Participants were asked to rate their health by responding to the statement: ‘Would you say that for someone of your age your own health in general is excellent, good, fair or poor?’ Participants reported the number of days each week they engaged in 30 min of physical activity using the single item measure validated by Milton et al. [23]. Next, participants were asked whether they had any responsibility for promoting health to employees working in their organisation and were also asked to select from 17 listed barriers, things which stopped them from participating in sport and physical activity (yes/no response options). The barriers were: not being active enough, time, being active not being a priority, not being the sporty type, poor health, having a long-term illness or disability, being overweight, being old, not being motivated, not being bothered, having no-one to do it with, cost, lack of facilities, lack of clubs or classes, having young children, fear of injury or ill health and other [24]. Finally, participants were asked to rate on a four-point scale from strongly agree to strongly disagree four statements relating to their employers support and provision for health and physical activity/sport. All statements began with the stem ‘My employer…’ and read: ‘promotes health and well-being’ and ‘promotes participation in sport and physical activity’. Response categories *strongly agree* and *agree* were later combined, as were *disagree* and *strongly disagree* to provide bivariate employer support variables for analysis.

#### Statistical analysis

Descriptive statistics were calculated using frequencies for categorical variables and mean ± 95 % confidence intervals (CI) for continuous variables. IBM SPSS Statistics 22 was used with alpha set at 0.05. Multinomial regression models were developed including demographic variables alongside variables which were hypothesised to influence knowledge of physical activity guidelines based on previous research. The nominal indicator of ‘accurate knowledge’ was assigned as the reference category. Evidence suggests that predictors of knowledge of physical activity guidelines differ between men and women [13] and so separate analyses were conducted for males and females. Variables found not to contribute to the prediction of the dependent variable were excluded from the final model.

##### Ethics, consent and permissions

All participants provided informed consent before taking part in this research.

## Results

A total of 10992 adults provided data on knowledge of the MVPA guideline and were included in the analysis. Individual and demographic characteristics are provided in Table 1. The sample was 37 % male, 95 % white and had a mean age of 38.9 ± 11 years. Over half (63 %) had a degree or higher and 81 % were in good or excellent health. A high proportion of respondents did not participate in sufficient physical activity to meet the recommended guideline (70.1 %). The proportion of respondents reporting barriers to participation in sport and physical activity is presented in Table 2 and perceived employer support for sport and physical activity in Table 3.

**Table 1:** Individual characteristics (n = 10,992), n and % unless otherwise stated

Characteristic	Number	Percent
Gender		
Male	4,120	37.5
Female	6,872	62.5
Age, years		
Mean ± 95 % CI	38.90	38.69, 39.10
Ethnic group		
Mixed	142	1.3
Black/Black British	177	1.6
Asian/Asian British	197	1.8
White British	10,357	95.3
Highest educational qualification		
Degree	6,967	63.4
Business and Technology Education Council (BTEC) Higher/Advanced Level (A Level)	2,332	21.2
BTEC National/General Certificate of Secondary Education (GCSE)	1,422	12.9
None/other	271	2.5
Job type		
Managerial e.g. office manager, finance manager	3,081	29.5
Professional e.g. nurse, teacher, police officer	4,127	39.5
Clerical/Admin e.g. secretary, office worker	2,799	26.8
Manual/Technical e.g. postal worker, farm worker	443	4.2
General health		
Excellent/Good	8,894	80.9
Fair/Poor	2,096	19.1
Physical activity engagement		
Meets guidelines (≥150 mins/week)	3,284	29.9
Doesn’t meet guidelines (<150 mins/week)	7,708	70.1

**Table 2:** Physical activity barriers (n = 10,992)

*What are the main things that stop you from participating in sport and physical activity*?	Number	Percent
I’m not active enough		
Yes	801	7.3
No	10,191	92.7
Time		
Yes	4,507	41
No	6,485	59
Being active is not a priority for me		
Yes	139	1.3
No	10,853	98.7
I’m not the sporty type		
Yes	917	8.3
No	10,075	91.7
My health is not good enough (n = 10,395)		
Yes	320	2.9
No	10,075	97.1
I have a long-term illness or disability		
Yes	274	2.5
No	10,718	97.5
I’m too overweight		
Yes	441	4
No	10,551	96
I’m too old		
Yes	131	1.2
No	10,861	98.8
I’m not motivated		
Yes	1,656	15.1
No	9,336	84.9
I can’t be bothered		
Yes	879	8
No	10,113	92
There is no-one to do it with		
Yes	1,024	9.3
No	9,968	90.7
Cost		
Yes	968	8.8
No	10,024	91.2
There are no suitable facilities		
Yes	304	2.8
No	10,688	97.2
There are no suitable clubs or classes		
Yes	387	3.5
No	10,605	96.5
I’ve got young children to look after		
Yes	1,438	13.1
No	9,554	86.9
I might get injured or damage my health		
Yes	259	2.4
No	10,733	97.6
Other*		
Yes	617	5.6
No	10,375	94.4

*most commonly reported other barrier was having a current injury

**Table 3:** Workplace-related predictors (n = 10,992)

	Number	Percent
I am responsible for promoting health at work (n = 10,985)		
Yes	1,976	18.0
No	9,009	82.0
My employer promotes health and wellbeing (n = 10900)		
Yes	8,953	82.1
No	1,947	17.9
My employer promotes participation in sport and physical activity (n = 10,850)		
Yes	7,669	70.7
No	3,181	29.3

The physical activity guideline was accurately reported by 15 % of adults while 13.8 % overestimated, 8.9 % underestimated and 62.3 % reported they did not know the guidelines (Table 4). No gender differences were found.

**Table 4:** Knowledge of physical activity guidelines, overall and by gender

Knowledge	Overall		Males		Females	
	n	%	n	%	n	%
Correct	1,642	15.0	572	14.0	1,070	15.7
Overestimate	1,504	13.8	524	12.8	980	14.3
Underestimate	970	8.9	340	8.3	630	9.2
Don’t know (no guess)	6,805	62.3	2654	64.9	4,151	60.8

Tables 5 and 6 present the odds ratios (OR), 95 % confidence intervals (CI) and p-values for knowledge of physical activity recommendations for males and females respectively. Analysis for the total sample is also provided in Additional file 1. Overestimation of the physical activity guideline within males was predicted by increasing age, not meeting recommendations for moderate-to-vigorous physical activity and not having personal responsibility to promote health at work. Overestimation within females was predicted by being of Black or Asian ethnicity, self-reporting poor health, reporting not being ‘sporty’ as a barrier to physical activity, having an employer who is perceived not to support physical activity and/or sport, not having personal responsibility to promote health at work and not meeting physical activity recommendations.

**Table 5:** Multinomial logistic regression model – predictors of knowledge of physical activity guidelines – male sample (n = 4,120)

		Overestimate	p-value	Underestimate	p-value	Don’t know	p-value
		OR [95 % CI]		OR [95 % CI]		OR [95 % CI]	
*Demographics*							
Age		1.02 [1.01, 1.03]	0.001*	1.01 [1.00, 1.03]	0.11	1.03 [1.02, 1.04]	0.00*
Ethnic group	Mixed	1.68 [0.59, 4.80]	0.33	1.16 [0.33, 4.09]	0.81	1.99 [0.85, 4.70]	0.11
	Black/Black British	0.62 [0.20, 1.91]	0.40	1.17 [0.40, 3.37]	0.78	0.81 [0.36, 1.81]	0.61
	Asian/Asian British	1.24 [0.44, 3.51]	0.68	1.29 [0.41, 4.05]	0.66	1.98 [0.89, 4.51]	0.10
	White British	Ref.	-	-	-	-	-
Education	Degree	0.49 [0.16, 1.51]	0.22	0.33 [0.11, 1.04]	0.06	0.37 [0.14, 0.96]	0.04*
	BTEC Higher/ALevel	0.78 [0.25, 2.53]	0.70	0.56 [0.17, 1.82]	0.34	0.90 [0.34, 2.40]	0.84
	BTEC National/GCSE	1.20 [0.34, 4.20]	0.78	0.97 [0.27, 3.52]	0.96	1.62 [0.56, 4.70]	0.38
	None/Other	Ref.	-	-	-	-	-
Job type	Managerial e.g. office manager, finance manager	1.41 [0.75, 2.63]	0.29	1.75 [0.81, 3.76]	0.15	0.71 [0.44, 1.15]	0.16
	Professional e.g. nurse, teacher, police officer	1.26 [0.67, 2.38]	0.47	1.79 [0.84, 3.8]	0.13	0.89 [0.55, 1.42]	0.61
	Clerical/Admin e.g. secretary, office worker	1.39 [0.68, 2.83]	0.37	2.00 [0.87, 4.59]	0.10	1.10 [0.64, 1.88]	0.73
	Manual/Technical e.g. postal worker, farm worker	Ref.	-	-	-	-	-
General health	Excellent/Good	0.81 [0.55, 1.18]	0.27	0.69 [0.45, 1.04]	0.75	0.64 [0.47, 0.87]	0.00*
	Fair/Poor	Ref.	-	-	-	-	-
Physical activity behaviour	Meets guidelines	0.73 [0.56, 0.95]	0.20	1.24 [0.93, 1.65]	0.15	0.68 [0.56, 0.84]	0.00*
	Doesn’t meet guidelines	Ref.	-	-	-	-	-
*Barriers to physical activity*							
Not active as a barrier	No	0.93 [0.48, 1.82]	0.84	0.71 [0.35, 1.44]	0.34	0.79 [0.46, 1.35]	0.38
	Yes	Ref.	-	-	-	-	-
Not sporty as a barrier	No	0.60 [0.25, 1.43]	0.25	0.36 [0.16, 0.85]	0.19	0.32 [0.16, 0.66]	0.00*
	Yes	Ref.	-	-	-	-	-
*Workplace*-*related predictors*							
Employer promotes health	No	0.87 [0.54, 1.38]	0.54	0.79 [0.48, 1.30]	0.36	1.05 [0.73, 1.51]	0.80
	Yes	Ref.	-	-	-	-	-
Employer promotes sport/physical activity	Yes	0.71 [0.47, 1.08]	0.11	0.63 [0.40, 1.00]	0.04*	0.71 [0.51, 1.00]	0.04*
	No	Ref.	-	-	-	-	-
Responsible for promoting health in the workplace	Yes	0.39 [0.29, 0.51]	0.00*	0.48 [0.34, 0.66]	0.00*	0.21 [0.17, 0.26]	0.00*
	No	Ref.	-	-	-	-	-

Reference category: Correctly reports physical activity guidelines. *statistically significant (p < 0.05)

**Table 6:** Multinomial logistic regression model – predictors of knowledge of physical activity guidelines – female sample (n = 6,872)

		Overestimate	p-value	Underestimate	p-value	Don’t know	p-value
		OR [95 % CI]		OR [95 % CI]		OR [95 % CI]	
*Demographics*							
Age		1.01 [1.00, 1.02]	0.21	1.00 [0.99, 1.01]	0.98	1.01 [1.00, 1.02]	0.04*
Ethnic group	Mixed	0.76 [0.34, 1.73]	0.52	0.46 [0.15, 1.42]	0.18	0.71 [0.37, 1.34]	0.29
	Black/Black British	2.67 [1.26, 5.67]	0.01*	1.78 [0.74, 4.39]	0.20	1.92 [0.96, 3.82]	0.06
	Asian/Asian British	3.55 [1.46, 8.63]	0.01*	4.03 [1.58, 10.27]	0.00*	3.73 [1.67, 8.33]	0.00*
	White British	Ref.	-	-	-	-	-
Education	Degree	0.71 [0.32, 1.58]	0.40	0.36 [0.17, 0.80]	0.01*	0.36 [0.19, 0.69]	0.00*
	BTEC Higher/ALevel	0.99 [0.43, 2.27]	0.98	0.52 [0.23, 1.16]	0.11	0.66 [0.34, 1.29]	0.23
	BTEC National/GCSE	1.35 [0.57, 3.17]	0.50	0.78 [0.34, 1.83]	0.57	1.03 [0.52, 2.05]	0.94
	None/Other	Ref.	-	-	-	-	-
Job type	Managerial e.g. office manager, finance manager	0.72 [0.37, 1.41]	0.34	0.64 [0.31, 1.35]	0.24	0.49 [0.28, 0.85]	0.12
	Professional e.g. nurse, teacher, police officer	0.65 [0.33, 1.26]	0.20	0.70 [0.34, 1.44]	0.33	0.47 [0.27, 0.82]	0.01*
	Clerical/Admin e.g. secretary, office worker	0.86 [0.44, 1.68]	0.66	0.79 [0.38, 1.64]	0.53	0.76 [0.44, 1.33]	0.34
	Manual/Technical e.g. postal worker, farm worker	Ref.	-	-	-	-	-
General health	Excellent/Good	0.70 [0.53, 0.91]	0.01*	0.74 [0.55, 1.00]	0.05	0.57 [0.46, 0.71]	0.00*
	Fair/Poor	Ref.	-	-	-	-	-
Physical activity behaviour	Meets guidelines	0.70 [0.57, 0.85]	0.00*	1.04 [0.83, 1.30]	0.76	0.65 [0.56, 0.77]	0.00*
	Doesn’t meet guidelines	Ref.	-	-	-	-	-
*Barriers to physical activity*							
Not active as a barrier	No	0.88 [0.60, 1.30]	0.53	0.86 [0.55, 1.34]	0.49	0.77 [0.55, 1.06]	0.11
	Yes	Ref.	-	-	-	-	-
Not sporty as a barrier	No	0.70 [0.51, 0.98]	0.04*	0.85 [0.58, 1.34]	0.40	0.75 [0.57, 0.99]	0.04*
	Yes	Ref.	-	-	-	-	
*Workplace*-*related predictors*							
Employer promotes health	No	0.94 [0.68, 1.30]	0.70	0.78 [0.54, 1.12]	0.18	0.85 [0.66, 1.11]	0.24
	Yes	Ref.	-	-	-	-	-
Employer promotes sport/physical activity	Yes	0.72 [0.53, 0.97]	0.03*	0.66 [0.47, 0.92]	0.02*	0.60 [0.47, 0.76]	0.00*
	No	Ref.	-	-	-	-	-
Responsible for promoting health in the workplace	Yes	0.5 [0.40, 0.62]	0.00*	0.52 [0.41, 0.66]	0.00*	0.29 [0.24, 0.34]	0.00*
	No	Ref.	-	-	-	-	-

Reference category: Correctly reports physical activity guidelines. *statistically significant (p < 0.05)

Underestimation of the physical activity guideline within males was predicted by reporting not being ‘sporty’ as a barrier to physical activity, having an employer who is perceived not to support physical activity and/or sport and not having personal responsibility to promote health at work. Underestimation within females was predicted by being of Asian ethnicity, low educational attainment, having an employer who is perceived not to support physical activity and/or sport and not having personal responsibility to promote health at work.

Reporting “don’t know” within males was predicted by increasing age, low educational attainment, having a technical/manual job instead of a managerial or professional job (nurse, teacher, police officer etc.), self-reporting poor health, reporting not being ‘sporty’ as a barrier to physical activity, having an employer who is perceived not to support physical activity and/or sport, not having personal responsibility to promote health at work and not meeting recommendations for moderate-to-vigorous physical activity. Reporting “don’t know” within females was predicted by increasing age, Asian ethnicity, low educational attainment, poor health, reporting not being ‘sporty’ as a barrier to physical activity, having an employer who is perceived not to support physical activity and/or sport, not having personal responsibility to promote health at work and not meeting physical activity recommendations.

## Discussion

Results indicated a low prevalence of knowledge of the MVPA guideline (15 %) within this sample of adults employed in England. Similar results have been reported within another sample of UK adults [12]. Both the Knox et al. sample and the present sample were highly educated relative to the population [12]. This research therefore, highlights the need for educational intervention even amongst highly educated and employed adults.

Similarly to Hunter et al. [13], the present study found distinct predictors of overestimation, underestimation and being unaware of physical activity recommendations. For instance, low education predicted overestimation and not knowing guidelines. Thus, less educated individuals may perceive an adequate amount of physical activity to be unattainable which could result in demotivation. We also found ethnicity to only predict overestimation, underestimation and not knowing the guideline within females. This suggests that certain female communities may require targeted promotions. Further examination of these factors is required.

The present study presents the first findings regarding the influence of employer support-related factors on knowledge of physical activity guidelines which is a determinant of physical activity behaviour [8, 9]. Lucove et al. [16] previously found that adults who perceived their workplace to have physical activity policies such as, on-site facility access and subsidies for health-clubs, were more likely to be engaging in physical activity than those who did not perceive their workplace to have such policies. The present findings suggest that employers could have a powerful influence on employee’s knowledge of physical activity guidelines. Lack of employer support for physical activity emerged as a predictor of overestimating, underestimating and not knowing the guideline, especially for females. More comprehensive investigation of these findings, including additional employer and workplace-related predictors in recognition of the likely complexity of this relationship is warranted. Employers could, however, play a role in educating their employees as to the current recommendations for participating in physical activity, as well as employing strategies such as role-modelling physical activity behaviour (i.e. being active themselves), rewarding active travel, initiating schemes to incentivise physical activity (e.g. collecting activity points which can be redeemed for prizes), sponsoring active breaks or encouraging walking meetings, providing an environment which supports physical activity e.g. installing lockers, bike sheds and showers etc., informing employees about active opportunities in the local area and advocating physical activity in regular workplace newsletters or bulletins. In addition, employees who highlighted themselves as being responsible for promoting health in the workplace were more likely to know the guideline. Thus, employers could utilise these members of staff to assist with educating other employees.

A further finding of the present research was that adults who reported ‘not being “sporty” enough’ as a barrier to engaging in physical activity were less likely to accurately report the physical activity guideline. On the other hand, reporting ‘not being active’ as a barrier to engaging in physical activity did not predict a lack of knowledge. The physical activity guidelines were designed to summarise evidence on the level of activity required to achieve health benefits. They may not be sufficiently accessible for individuals of all activity levels and may be failing to inspire the least active to engage in physical activity by encouraging generally active lifestyles, which does not have to be achieved through sport [2]. It is possible that some individuals are unable to differentiate between physical activity and sport and this could also be impeding accurate health knowledge. This highlights the need for public facing messages to be developed to aid communication of the physical activity guidelines and increase understanding of the levels of participation required, as well as the steps that can be taken to achieve these levels. It may be beneficial for workplaces to promote physical activity guidelines, within general wider promotional efforts towards a more active lifestyle. For instance, workplaces could encourage taking ten minute walk breaks at lunch as a way of accumulating the recommended 150 min a week of MVPA.

The findings reported in the present study are cross-sectional in nature and so cannot be used to infer causality requiring cautious interpretation. There are also a number of limitations to online survey research such as lack of control over the test environment and inability to measure sampling frame. Further, some of the measures employed have not been reliability tested and our sample was both highly educated, more likely to be employed and less representative of ethnic minorities relative to the general population. Finally, as data was collected through the *County Sports Partnership Network Workplace Challenge* there may have also been a degree of selection bias, though it must be noted that knowledge of the MVPA guideline was still low. As the first study to assess knowledge of guidelines in a large sample of adults working in England and to specifically consider employer support-related influences we believe the results can still provide useful insights into this targeted population.

## Conclusions

The present study identified a low prevalence of knowledge of the MVPA guideline within employed adults in England. Employers have a potential role to play in educating employees in relation to the current physical activity guidelines and those who actively promote and support involvement in physical activity can contribute significantly to better health knowledge and so should be targeted as important sources of social influence.

## Additional file

Additional file 1:
**Table A1.** Multinomial logistic regression model – predictors of knowledge of physical activity guidelines – total sample (N = 10992).
