# Supplementary material for: Knowledge of physical activity recommendations in adults employed in England: associations with individual and workplace-related predictors
Source: Int J Behav Nutr Phys Act. 2015 May 23;12:69. doi: 10.1186/s12966-015-0231-3 (PMC4445563; doi:10.1186/s12966-015-0231-3)
Supplement: Additional file 1: — Table A1. Multinomial logistic regression model – predictors of knowledge of physical activity guidelines – total sample (N = 10992). [file 12966_2015_231_MOESM1_ESM.docx]

**Additional files**

**Additional file 1.**

**Table A1** Multinomial logistic regression model – predictors of knowledge of physical activity guidelines – total sample (N=10992)

|  | | Overestimate | Underestimate | Don’t know |
| --- | --- | --- | --- | --- |
|  |  | OR [95% CI] | OR [95% CI] | OR [95%] |
| Age |  | 1.01 [1.00, 1.02] | 1.00 [1.00, 1.01] | 1.01 [1.01, 1.02] |
| Gender | Female | 0.93 [0.80, 1.09] | 0.92 [0.77, 1.10] | 0.70 [0.61, 0.79] |
|  | Male | Ref. | Ref. | Ref. |
| Ethnicity | Mixed | 1.03 [0.54, 1.94] | 0.68 [0.30, 1.54] | 1.07 [0.65, 1.77] |
|  | Black/Black British | 1.70 [0.94, 3.08] | 1.51 [0.76, 2.97] | 1.41 [0.84, 2.36] |
|  | Asian/Asian British | 2.32 [1.21, 4.45] | 2.53 [1.26, 5.08] | 2.79 [1.59, 4.89] |
|  | White British | Ref. | Ref. | Ref. |
| Highest educational attainment | Degree | 0.63 [0.33, 1.21] | 0.35 [0.19, 0.68] | 0.37 [0.21, .62] |
|  | BTEC Higher/A Level | 0.92 [0.47, 1.81] | 0.53 [0.27, 1.03] | 0.74 [0.43, 1.28] |
|  | BTEC National/GCSE | 1.26 [0.62, 2.54] | 0.81 [0.40, 1.65] | 1.16 [0.65, 2.06] |
|  | None/Other | Ref. | Ref. | Ref. |
| General health | Excellent/Good | 0.73 [0.59, 0.91] | 0.72 [0.56, 0.92] | 0.60 [0.50, .71] |
|  | Fair/Poor | Ref. | Ref. | Ref. |
| Job type | Manager | 1.02 [0.65, 1.61] | 1.06 [0.63, 1.78] | 0.60 [0.42, 0.86] |
|  | Professional | 0.91 [0.58, 1.42] | 1.12 [0.66, 1.84] | 0.64 [0.45, 0.91] |
|  | Clerical/Admin | 1.14 [0.72, 1.82] | 1.24 [0.74, 2.10] | 0.94 [0.65, 1.35] |
|  | Technical/Manual | Ref. | Ref. | Ref. |
| Physical activity behaviour | Meets guidelines | 0.71 [0.60, .83] | 1.10 [0.93, 1.32] | 0.66 [0.58, 0.75] |
|  | Doesn’t meet guidelines | Ref. | Ref. | Ref. |
| Not active enough as a barrier to physical activity | No | 0.89 [0.64, 1.24] | 0.82 [0.56, 1.19] | 0.78 [0.59, 1.02] |
|  | Yes | Ref. | Ref. | Ref. |
| Not sporty enough as a barrier to physical activity | No | 0.69 [0.51, .94] | 0.74 [0.52, 1.05] | 0.65 [0.51, 0.84] |
|  | Yes | Ref. | Ref. | Ref. |
| Employer promotes sport/physical activity | Yes  No | 0.70 [0.55, 0.90]  Ref. | 0.64 [0.49, 0.83]  Ref. | 0.62 [0.51, 0.76]  Ref. |
| Employer promotes health | No | 0.90 [0.69, 1.18] | 0.77 [0.57, 1.03] | 0.91 [0.73, 1.12] |
|  | Yes | Ref. | Ref. | Ref. |
| Responsible for promoting health in the workplace | Yes | 0.46 [0.39, 0.54] | 0.50 [0.42, 0.61] | 0.26 [0.22, 0.29] |
|  | No | Ref. | Ref. | Ref. |

Reference category: Aware of physical activity guidelines.
